# “Go To Travel” Campaign and Travel-Associated Coronavirus Disease 2019 Cases: A Descriptive Analysis, July–August 2020

**DOI:** 10.3390/jcm10030398

**Published:** 2021-01-21

**Authors:** Asami Anzai, Hiroshi Nishiura

**Affiliations:** Kyoto University School of Public Health, Yoshida-Konoe-cho, Sakyo-ku, Kyoto 606-8501, Japan; anzai.asami.2x@kyoto-u.ac.jp

**Keywords:** tourism, sightseeing, epidemiology, severe acute respiratory syndrome coronavirus 2 (SARS-CoV-2), coronavirus disease 2019 (COVID-19), mobility

## Abstract

The Japanese government initiated the Go To Travel campaign on 22 July 2020, offering deep discounts on hotel charges and issuing coupons to be used for any consumption at travel destinations in Japan. In the present study, we aimed to describe the possible epidemiological impact of the tourism campaign on increasing travel-associated cases of coronavirus disease 2019 (COVID-19) in the country. We compared the incidence rates of travel-associated and tourism-related cases prior to and during the campaign. The incidence of travel-associated COVID-19 cases during the tourism campaign was approximately three times greater than the control period 22 June to 21 July 2020 and approximately 1.5 times greater than the control period of 15 to 19 July. The incidence owing to tourism was approximately 8 times and 2–3 times greater than the control periods of 22 June to 21 July and 15 to 19 July, respectively. Although the second epidemic wave in Japan had begun to decline by mid-August, enhanced domestic tourism may have contributed to increasing travel-associated COVID-19 cases during 22 to 26 July, the early stage of the Go To Travel campaign.

## 1. Introduction

As of 1 November 2020, Japan had recorded a total of 99,959 confirmed cases of coronavirus disease 2019 (COVID-19), including 1765 deaths [1]. Owing to widespread domestic transmission, a state of emergency was declared on 7 April 2020 in seven prefectures (Tokyo, Saitama, Chiba, Kanagawa, Osaka, Hyogo, and Fukuoka); this was expanded to all 47 prefectures on 16 April 2020. The incidence of COVID-19 subsequently decreased over time [2], and the state of emergency was gradually lifted on 14 May, and had been lifted in all prefectures by 25 May 2020. Following the end of the first wave, a resurgence of COVID-19 cases was seen from late June in urban prefectures, most notably in Tokyo and Osaka; the reported number of confirmed cases reached 1595 cases per day on 7 August 2020. In response, local prefectural governments implemented focused interventions to reduce disease transmission in high-risk settings, including nightclubs, bars, and restaurants. The incidence of COVID-19 peaked between late July and early August in many prefectures; the prevalence of hospital admissions peaked on 10 August with 13,724 cases and then declined. Presently, Japan is facing a third epidemic wave, with a marked resurgence in the number of COVID-19 infections beginning in early October 2020 [1]. 

The impact of COVID-19 has affected various industrial and social sectors, most notably the tourism sector, including hotels, lodging, and transportation. The Japan Tourism Agency regularly conducts the Overnight Travel Statistics Survey [3] and has estimated that the relative number of hotel guests staying in business hotels, resort hotels, city hotels, and ryokan (a classical Japanese style lodging) from March to June in 2020 decreased by 48.9–84.9% compared with the same period in 2019. Moreover, the total number of foreign visitors to Japan was 32 million in 2019, but the cumulative count by October 2020 was only 4 million [4]. As an economic countermeasure to rescue adversely affected sectors by boosting consumer activities, the Japanese government initiated the “Go To Travel” campaign on 22 July 2020, slightly ahead of its originally scheduled start date (early August 2020). July 22 was the day before the 4-day holiday from 23 to 26 July. The campaign offered drastic discounts on hotel charges and issued coupons that acted as a “cash back” system and that could be used for any type of consumption at travel destinations, thereby increasing consumer demand with the aim of stimulating economic activity at local levels [5]. Similarly, other countries have promoted domestic travel in various ways [6].

Enhancing human mobility can facilitate the transmission of COVID-19. There was a concern among all involved prefectures to run the campaign [7]. Because the epidemic activity in Tokyo was intense during late July 2020, any travel to Tokyo was excluded from the Go To Travel campaign, and residents of Tokyo were not permitted to enjoy the benefits of the campaign [8]. In fact, the governor of Tokyo requested that Tokyo residents practice self-restraint and refrain from traveling outside the city; this was announced shortly before and after the Go To Travel campaign (e.g., the governor’s statement on 15 July [9]). Moreover, Okinawa Prefecture experienced a surge of COVID-19 cases and the governor declared a state of emergency on 1 August 2020, requesting that Okinawa residents do not leave the city and for other travelers to reconsider visiting Okinawa [10]. In mid-August, there is the Obon holiday season in Japan, during which many people visit their hometowns. Prior to Obon, most prefectures warned their residents and prospective visitors to carefully decide whether or not to visit their hometowns, especially when feeling unwell [11].

To understand the potential drawbacks of the Go To Travel campaign, epidemiological analysis of travel-associated transmission dynamics is imperative. The potential effects of enhancing mobility can range from increased infections among travelers to enhanced virus transmission at local levels. As a first evaluation attempt, in the present study, we focused on the epidemiological patterns of travel-associated cases of COVID-19 in remote prefectures of Japan. We aimed to describe the possible impact of the Go To Travel campaign on increasing travel-associated cases of COVID-19 infection caused by increased mobility across prefectural borders in Japan. 

## 2. Methods

### 2.1. Epidemiological Data

The present study was a descriptive epidemiological study using publicly available data (surveillance data). We scanned all press release datasets of confirmed COVID-19 cases reported by prefectural governments in Japan. Among the total 47 Japanese prefectures, 24 prefectures consistently provided information regarding mobility patterns across prefectural borders for each confirmed case. That is, travel information for patients with confirmed COVID-19 was reported by those local governments if he/she had a history of travel within 7 days (within 14 days in some prefectures) prior to disease onset. We retrieved these case counts prior to and during implementation of the Go To Travel campaign.

### 2.2. Scope of Descriptive Analysis

We analyzed COVID-19 cases reported by prefectural governments from 1 May to 31 August 2020. The latest time that we screened the press release was mid-November 2020. To understand the impact of the Go To Travel campaign, the possible date of infection rather than the date of reporting for cases was considered when grouping and comparing those who may have been infected before and during the campaign. To infer the possible date of infection, we had two sets of cases for analysis: (i) all cases with a known date of illness onset and (ii) all cases with a known date of COVID-19 confirmation. We subtracted 5 days (= the mean incubation period [12]) from the date of illness onset and 10 days (= the sum of mean incubation period and mean time from illness onset to report [13]) from the date of confirmation such that the calendar date of the respective cases was converted to the assumed date of infection. Average turnaround time of polymerase chain reaction testing was 1 day, and this was included as part of the time delay from illness onset to reporting.

According to contact history, cases were grouped by movement history across prefectural borders prior to illness onset; hereafter, travel-associated cases refer to those individuals that crossed prefectural borders. If the travel history was known, travel-associated cases were further classified according to the purpose of travel (i.e., business, family visit, or tourism/sightseeing). Travel-associated cases included those individuals who themselves had crossed prefectural borders and those who had contact with someone who had done so. 

### 2.3. Statistical Analysis

The incidence of travel-associated cases during the Go To Travel campaign was compared against the incidence before the start of the campaign, using the incidence rate ratio (IRR). The IRR is the ratio of incidence, comparing two periods of time, and the value above 1 indicates that the incidence during Go To Travel campaign increased compared with the baseline period. Specifically, the IRR was calculated according to travel history, and we also calculated the IRR of cases involving travel for tourism purposes, which was the aim of the tourism campaign. The treatment period was defined as 22–26 July (5 days), where 22 July was the first date of the campaign, coinciding with a 4-day holiday from 23 to 26 July 2020. Hereafter, we refer to this period as Period 2. We considered two different time periods as our controls: (i) 22 June to 21 July, the 30 days (Period 1a) prior to the campaign, and (ii) 15–19 July, a 5-day period (Period 1b) prior to the campaign, set to include the same days of the week as Period 2. Although the Go To Travel campaign continued into August, the campaign involved a fixed budget and the campaign ceased once the budget ceiling was reached. In the case of the first campaign from 22 July, the deadline for coupon redemption was set to 31 August 2020. The Obon festival holiday is celebrated in Japan in mid-August; therefore, we also examined the incidence from 8 to 31 August 2020 (24 days) as Period 3 for a comparison to the later stage of the tourism campaign.

### 2.4. Ethical Considerations

In the present study, we analyzed publicly available data, which had been de-identified upon press release. The present study was approved by the Medical Ethics Board of the Graduate School of Medicine, Kyoto University (R2673).

### 2.5. Data Sharing Statement

Summary data of cases, with and without travel history, are available Summary data of cases, with and without travel history, are available in Table S1 and S2.

## 3. Results

A total of 3978 confirmed COVID-19 cases were reported from 1 May to 31 August 2020 in 24 prefectures of Japan. Male individuals accounted for 57.3% of cases (2211 cases); the sex was unknown for 119 cases. The average patient age was 42.6 years. The presence of symptoms upon diagnosis was known for 3060 patients (76.9% of the total), involving 2150 mild cases (70.3% of cases with known symptoms) and 891 asymptomatic cases (29.1%).

**Figure 1:**
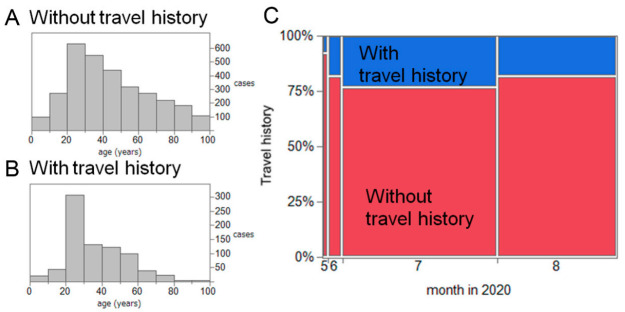
Age and time distributions for confirmed cases (*n* = 3978) of coronavirus disease 2019 (COVID-19) in 24 prefectures of Japan with consistent reporting of travel history from 1 May to 31 August 2020. (**A**,**B**). Age distribution of cases with and without travel history information (*n* = 3161 and *n* = 817, respectively). (**C**). Monthly count of confirmed cases, by travel history. Colored areas depict corresponding relative frequencies of cases.

Of the 3978 cases, 817 (20.1%) had a travel history across prefectural borders or a contact history with another person who crossed prefectural borders; we defined these as travel-associated cases. The average age of travel-associated cases was 36.2 years old, and that of the remaining cases was 44.2 years old (Figure 1A). The month of reporting for all confirmed cases is shown in Figure 1B, dominated by July with 2074 cases (52.7%), followed by August with 1597 cases (40.5%). There were 482 travel-associated cases (23.2%) in July and 289 cases (18.1%) in August 2020.

**Figure 2:**
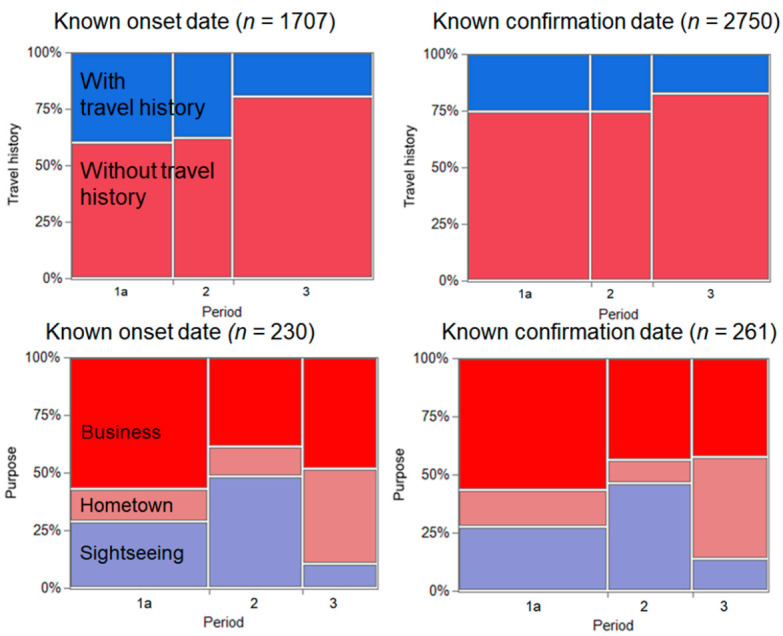
Comparison of coronavirus disease 2019 (COVID-19) incidence before and during the Go To Travel campaign. (**Left**) Cases with known illness onset and (**Right**) cases with known date of COVID-19 confirmation. (**Top**) Comparison by travel history and (**Bottom**) by purpose of travel. Period 1 corresponds to Period 1a (22 June to 21 July 2020). Periods 2 and 3 correspond to 22–26 July and 8–31 August 2020.

We compared the COVID-19 incidence across Period 1a (from 22 June to 21 July), Period 2, and Period 3 according to travel history and purpose of travel (Figure 2). In total, there were 1707 and 2750 confirmed cases with a known date of illness onset and date of COVID-19 confirmation, respectively. When using the known date of illness onset, the IRR of Period 2 was 3.31 (95% confidence interval (CI): 2.67–4.11) in comparison with Period 1a. Similarly, when using the date of confirmation, the IRR was estimated to be 2.98 (95% CI: 2.43–3.65). 

Focusing on the purpose of travel, we identified that tourism increased during the Go To Travel campaign. There were 30 and 34 confirmed COVID-19 cases with known dates of illness onset during Periods 1a and 2, respectively. Similarly, there were 35 and 33 confirmed cases with known dates of COVID-19 confirmation in Periods 1a and 2, respectively. Accordingly, the IRR was estimated as 6.80(95% CI: 4.16–11.11) and 5.66 (95% CI: 3.52–9.10), respectively, for known dates of illness onset and confirmation.

**Figure 3:**
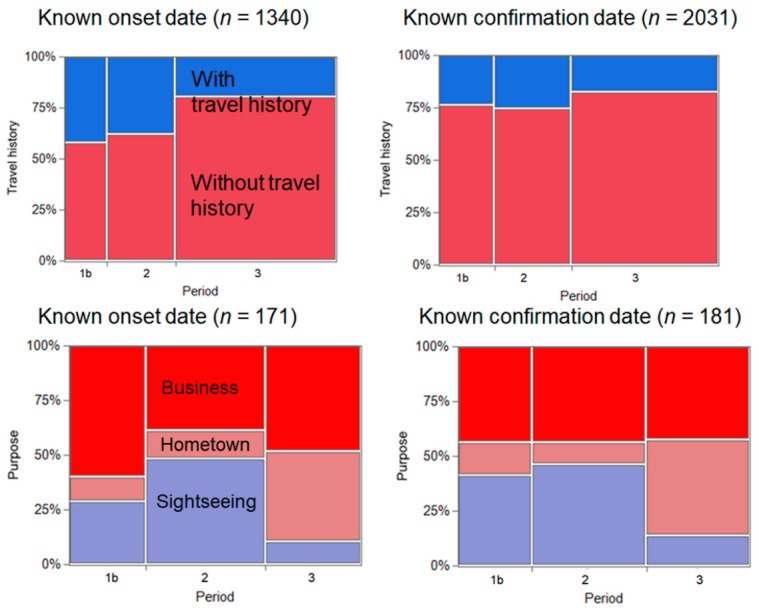
Comparison of coronavirus disease 2019 (COVID-19) incidence before and during the Go To Travel campaign. (**Left**) Cases with known illness onset and (**Right**) cases with known date of confirmation. (**Top**) Comparison by travel history and (**Bottom**) by purpose of travel. Period 1 corresponds to Period 1b (15 July to 19 July). Periods 2 and 3 correspond to 22–26 July and 8–31 August 2020.

Figure 3 shows a comparison of the incidence of COVID-19 across Period 1b (from 15 to 19 July), Period 2, and Period 3, according to travel history and purpose of travel. In total, there were 1340 and 2031 confirmed cases with a known date of illness onset and date of COVID-19 confirmation. When using the known date of illness onset, the IRR of Period 2 was 1.44 (95% CI: 1.10–1.89) compared with Period 1b. Similarly, when using the date of confirmation, the IRR was estimated to be 1.48 (95% CI: 1.14–1.93). There were 13 and 34 travel-related cases with known dates of illness onset in Periods 1b and 2, respectively; the IRR was estimated as 2.62 (95% CI: 1.38–4.96). In Periods 1b and 2, there were 19 and 33 confirmed cases of COVID-19 with known dates of confirmation, respectively, and the IRR was estimated as 1.74 (95% CI: 0.99–3.05).

## 4. Discussion

In the present study, we analyzed surveillance-based datasets containing information of travel-associated cases of COVID-19 infection, prior to and during the Go To Travel campaign, and we compared the incidence rate of travel-associated cases and those travelling for tourism purposes. The incidence of travel-associated cases during the campaign appeared to be approximately three times greater than that during control period 1a (from 22 June to 21 July) and approximately 1.5 times greater than the incidence during control period 1b (from 15 to 19 July). Notably, the incidence related to tourism was approximately eight times greater than that during control period 1a and 2–3 times greater than the incidence during control period 1b. Although the second epidemic wave in Japan had begun to decline by mid-August, the number of travel-associated cases increased during the Go To Travel campaign. 

To the best of our knowledge, the present study is the first to demonstrate that the number of travel-associated COVID-19 cases that involved crossing of prefectural borders increased during the Go To Travel campaign. For both control periods 1a and 1b, and also for both datasets with known dates of illness onset and COVID-19 confirmation, we showed that the IRR clearly exceeded a value of 1. In particular, the present study findings demonstrated that travel-associated cases related to tourism increased markedly in terms of proportion and incidence during the campaign. Enhanced domestic tourism may have contributed to increasing the number of travel-associated cases of COVID-19 in Japan, at least during Period 2, the early part of the Go To Travel campaign. 

Our descriptive analysis was too simplified to be able to conclusively determine a causal relationship between the tourism campaign and COVID-19 incidence in Japan. In particular, it must be acknowledged that the Go To Travel campaign started during a 4-day holiday period. Thus, the enhanced number of tourists traveling throughout the country cannot be attributed solely to the campaign. Another issue to be noted is that the severity of the epidemic was increasing in urban prefectures during the periods examined, such as in Tokyo and Osaka, which were not included in our analysis [14,15]. To address the potential trend in the 24 included prefectures and further clarify any causal effect, we are conducting another series of investigations using a quasi-experimental study design with time-series data, including a difference-in-differences study and interrupted time-series analysis [16]. In the mean time, a policy working paper was published, suggesting that the campaign increased the number of hotel guests without intensifying the spread of infection [17]. We will also analyze data from another round of the Go To Travel campaign that began on 1 October 2020. The present study is intended to be a rapid report, including a descriptive analysis of incidence data, to provide further insight into the potential impact of the tourism campaign on epidemic dynamics. During the summer of 2020, a campaign called “Eat Out to Help Out” was conducted in the United Kingdom with the aim of supporting the food service industry [18]. That campaign was not intended to promote domestic travel, but it has been reported that the campaign may have affected local transmission in the United Kingdom [19].

Although the policy decision to conduct the Go To Travel campaign was made on the basis of decreased epidemic activity and the expected positive impact of the campaign on socioeconomic activities, it is natural that enhancing human mobility across wider geographic areas would facilitate additional contact and thus increase the spatiotemporal spread of disease [20,21]. When the state of emergency was lifted in Japan around the middle of May (i.e., 21 May for Osaka and 25 May for Tokyo), the original plan was for the reduction in travel restrictions to begin in August, and the campaign was originally intended to start during that month [22]. However, the campaign schedule was moved forward, even as cases were increasing in Tokyo and Osaka and the country was trying to regain control of the epidemic. Government policies have had to strike a balance between epidemic control and restoration of economic activities, and managing both was the justification for implementing the tourism campaign [23]. Thus, experts in infectious diseases and public health cooperated with the government, formulating guidelines for infection control in each sector and offering advice to maximally reduce infections via the use of precautionary behaviors (e.g., wearing masks, avoiding close contact in confined spaces, and hand hygiene). In fact, the campaign included guidance on preventive measures for both travelers and service providers [24]. Nevertheless, the present study findings indicated that these efforts were accompanied by increased travel-associated cases of COVID-19 infection.

Four limitations in this study should be acknowledged. First, our datasets involved reporting bias that may have varied by prefecture. Although the government has announced the essential policy regarding their disclosure of information over diagnosed cases [25], the extent of information disclosure has varied widely according to prefecture. For example, the Tokyo Metropolitan Government does not disclose detailed information such as travel history for individual cases [26]. In addition, epidemic size has differed among prefectures (e.g., urban prefectures with many cases have had greater difficulty identifying travel-associated cases than remote prefectures with small numbers of cases), and rural areas have tended to report cases more frequently than urban ones. Second, the surveillance data relied on cases of COVID-19 infection confirmed using reverse transcriptase polymerase chain reaction, and these data involve ascertainment bias [27]. Considering that younger people are more mobile than older ones and the severity of infection among young adults is limited compared with that among older people, the absolute risk of travel may have been underestimated. Third, in addition to the direct impact of the tourism campaign, its possible indirect impact (e.g., enhanced contact induced by easing of travel restrictions) should be further explored. Fourth, the epidemiological impact of the tourism campaign has not been fully quantified. For instance, increased local clusters owing to travel should be examined, and the occurrence of epidemic waves in remote prefectures could also indicate the epidemiological outcome of the campaign.

Although numerous future tasks remain, we believe that the present study provides critical insights into the epidemiological impact of the Go To Travel campaign on the transmission dynamics of COVID-19 in Japan. To identify policy measures that can balance epidemic control and restoration of economic activity, additional evidence is critically needed.

## Data Availability

Data is contained within the supplementary material: The data presented in this study are available in Tables S1 and S2.

## Supplementary Materials

The following are available online at https://www.mdpi.com/2077-0383/10/3/398/s1. Table S1: Nubmer of cases with and without travel history by defined time periods. Table S2: Nubmer of cases with travel history and with known purpose of travel.
